# The Relationship Between Vitamin D Levels and Severity in Illness in COVID-19 Patients: A Cross-Sectional Study

**DOI:** 10.7759/cureus.23146

**Published:** 2022-03-14

**Authors:** Nirav Nimavat, Shruti Singh, Divyang Patel, Pratibha Singh, Mohammad Mehedi Hasan, Gowthamm Mandala, Ranvir Bhangu, Aakanksha Priya

**Affiliations:** 1 Community Medicine, Pacific Institute of Medical Sciences, Udaipur, IND; 2 Pharmacology, All India Institute of Medical Sciences Patna, Patna, IND; 3 Community Medicine, Dr. N. D. Desai Faculty of Medical Science and Research, Nadiad, IND; 4 Anaesthesiology, Sanjay Gandhi Postgraduate Institute of Medical Sciences, Lucknow, IND; 5 Biochemistry and Molecular Biology, Mawlana Bhashani Science and Technology University, Tangail, BGD; 6 Biological Sciences, Center Groove High School, Greenwood, USA; 7 Medicine and Surgery, Caribbean Medical University, Des Plaines, USA

**Keywords:** vitamin d supplementation, low vitamin d, vitamin-d deficiency, vitamin d level, covid 19

## Abstract

Introduction: The coronavirus disease 2019 (COVID-19) pandemic hit the world badly with high mortality. Severe acute respiratory syndrome coronavirus 2 (SARS-CoV-2) infection increased the COVID-19 burden among developed and developing countries due to the unavailability of proven treatment options. Vitamin D has many important anti-inflammatory, immunomodulator, and anti-viral functions. The present study was conducted to evaluate the relationship between Vitamin D in COVID-19.

Methods: A cross-sectional study was conducted at a tertiary care hospital in Patna, India. All the patients were enrolled during the period of 3.5 months. A chemiluminescence-based immunoassay analyzer was used to quantify Vitamin D among COVID-19 patients. The study compared Vitamin D deficiency and insufficiency among different groups, i.e., age, sex, BMI, comorbidity, etc. Diabetes and hypertension were evaluated as risk factors for mortality.

Results: A total of 225 patients were investigated. Of these, 13.6% had Vitamin D deficiency and 38.9% had insufficiency. Vitamin D level was statistically significant among different age groups, sex, and smokers. Patients aged >60 years were 23 times more likely to have a severe illness (adjusted OR (aOR) 23.53, 95%CI 4.67-118.61), whereas those aged 40 to 60 years were 11 times more likely to have a severe illness (aOR 10.86, 95%CI 2.39-49.31). Patients with many comorbidities, on the other hand, had a tenfold greater chance of severe COVID-19 (aOR 9.94, 95%CI 2.47-39.88). A deficiency of vitamin D increased the chance of a serious illness by nearly five times (aOR 4.72, 95%CI 1.31-17.03).

Conclusion: Vitamin D level was associated with severity of illness; it can be used to estimate the prognosis of COIVD-19 patients and aid in the modification of treatment protocols.

## Introduction

In December 2019, the World Health Organization (WHO), China Country Office, received news of a pneumonia epidemic in Wuhan, China, with an unknown etiology. Within a month or so, more cases of this novel coronavirus were identified in various countries worldwide. In March 2020, the WHO declared coronavirus disease 2019 (COVID-19) a pandemic [1]. The virus had infected at least 418 million worldwide as of February 20, 2022. Furthermore, it has cost the lives of 5.8 million people worldwide [2]. Due to the lack of a viable treatment, health experts worldwide were left with no therapeutic choices, and vaccines were designed with effectiveness and protection in mind; the virus continued to spread, infecting and killing more people.

Vitamin D is essential for immune system enhancement. We have witnessed a significant shift in our understanding of Vitamin D's medicinal benefits during the last several years [3]. The typical role of Vitamin D as an element of mineral breakdown and other diseases related to bones has been extended to contain a more extensive job for homeostasis and common bone problems like osteoporosis. In any case, it is the non-skeletal role of Vitamin D that has managed to pull in most consideration. Although much feedback regarding this vitamin has been available for a long time, our viewpoint on the non-classical capacity of Vitamin D comes from a few latest notions [4]. Firstly, we can consider the deficiency of Vitamin D among the majority of the population worldwide. This point has incited studies to investigate the effect of an imperfect or lower level of Vitamin D, linking it with many other severe health conditions like autoimmune disorders, cardiovascular disease (CVD), hypertension (HTN), and other diseases. Similarly, various studies have been conducted on the positive benefits of Vitamin D on the early recovery and prevention of COVID-19 [5,6].

COVID-19 is a systemic, respiratory disorder. The occurrence of pneumonia dictates the seriousness of COVID-19, severe Acute respiratory distress syndrome (ARDS), myocarditis, microvascular thrombosis, and additionally hypercytokinemia, each of these causing acute inflammatory reactions. The main savior against aggravated inflammation, including any infection by a virus in general, is T lymphocyte. The level of T lymphocytes is low in many patients of COVID-19. It is found to be increased by the intake of Vitamin D. Vitamin D deficiency has also been linked to an expansion in inflammation-causing cytokines that increase the risk of pneumonia or upper respiratory tract infection [7].

Vitamin D shows a significant role in increasing the expressions of angiotensin-converting enzyme 2 (ACE2). ACE2 is a significant receptor that facilitates the process of pathogenesis in COVID-19. Vitamin D may also increase the countenance of genes related to oxidation. It can modify the adaptive immune response. Moreover, it can increase cellular immune response as well [8]. A study carried out in Spain found that patients administered with Vitamin D had a very low ICU admission rate compared to the non-intervention group [9]. Many researchers suggest that taking Vitamin D might not prevent SARS-CoV-2 from infecting but may reduce the symptoms to the minimum that it goes unnoticed, and the person never gets tested. Another study found that persons with low vitamin D levels were more likely to have respiratory failure due to SARS-CoV-2 [10]. On the contrary, two trials suggested that when given to seriously ill patients without COVID-19, Vitamin D did not cause any positive effects as compared to the patients treated with placebo [11]. The present study was carried out to evaluate the relationship between COVID-19 and Vitamin D.

## Materials and methods

**Figure 1:**
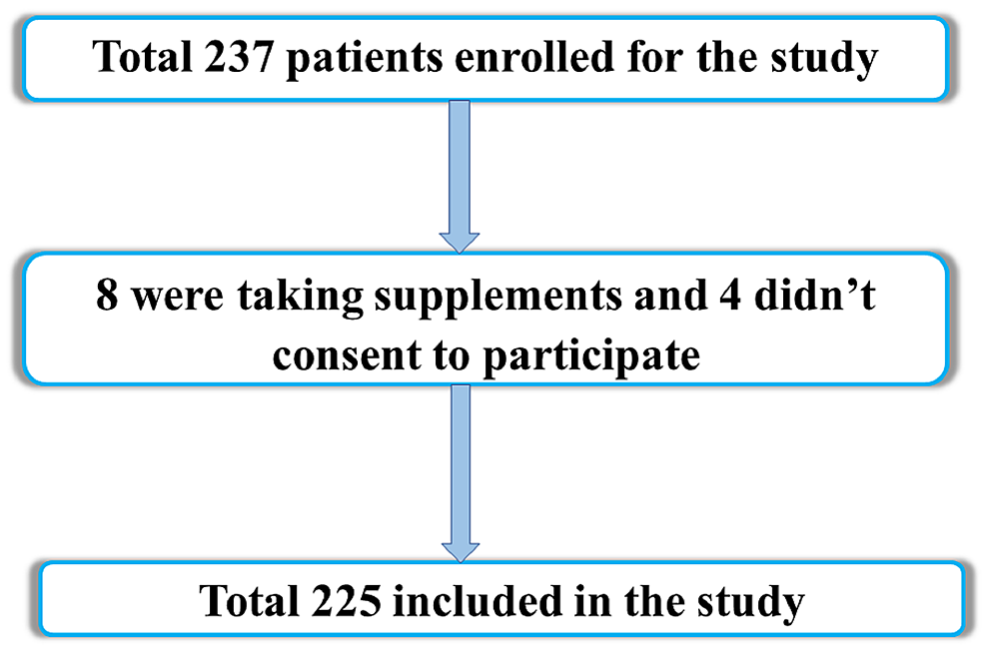
Enrolment and selection of subjects in the study

The study was conducted at a COVID-19 tertiary care hospital, the All India Institute for Medical Sciences (AIIMS), Patna, India. As there was no previous research available, sample size calculation was not feasible. So all COVID-19 patients, from August 1 to November 15, 2020, were enrolled for the study. Those who tested positive for SARS-CoV-2 at the Flu clinic and COVID-19 ward through reverse transcription-polymerase chain reaction (RT-PCR) were enrolled. Those who were using or had taken Vitamin D supplements in the preceding six months were exempted. The research's purpose was described to all patients, and written/digital consent was acquired to participate in the study. The institutional ethics committee of AIIMS, Patna, approved the research protocol dated July 10, 2020 (reference no AIIMS/Pat/IEC/2020/601). The study was conducted per the Helsinki Declaration. Data was collected using a pre-validated case recording form. The number of patients enrolled and selected for the study is shown in Figure 1.

Demographic information of all COIVD-19 patients was gathered. Diabetes, HTN, and other comorbid conditions data were collected. Consumption of alcohol and cigarette were taken into consideration during data collection. Vitamin D samples were collected by trained hospital staff and sent along with regular investigations. There was no issue of bias as the sample collection was blinded. The amount of Vitamin D was determined using the ADVIA Centaur® XP chemiluminescence-based immunoassay analyzer (Siemens Healthineers AG, Erlangen, Germany). All the data were entered into Microsoft Excel 365 (Microsoft Corp., Redmond, Washington, United States), and IBM SPSS Statistics for Windows, Version 27.0 9Released 2020. IBM Corp., Armonk, New York, United States) was used to analyze it. All of the demographic information was analyzed and presented in frequencies and percentages. The degree of vitamin D insufficiency, as well as the BMI, were all categorized. It was determined that a p-value of less than 0.05 was statistically significant. To further understand the Vitamin D status of the various groups, deficiency and insufficiency of Vitamin D were assessed independently and compared. Data on clinical severity was analyzed to determine a link between clinical severity, Vitamin D level, and age. Age, sex, Vitamin D, diabetes, and HTN were compared for mortality.

Patients were categorized based on their Vitamin D levels. Vitamin D levels >30 ng/mL were considered normal. Vitamin D insufficiency and deficiency were defined by blood values of 20-30 ng/mL and 20 ng/mL, respectively [12]. The patient's clinical severity was categorized into three groups. The severity was classified using guidelines by the Ministry of Health and Family Welfare, Government of India [13]. The patient's classification is based on the following clinical criteria: (1) Mild case: There are no symptoms of shortness of breath or hypoxia (normal saturation); (2) Moderate case: Dyspnea, hypoxia, fever, cough, SpO2 94% (range 90-94%) on room air, respiratory rate ≥ 24 per minute are all clinical features to look for; (3) Severe case: Pneumonia symptoms plus one of the following: breathing rate >30 breaths/min, significant respiratory distress, and SpO2 less than 90% on room air.

Obesity was classified following the WHO's recommendations for the Asia-Pacific area [14]. The conditions are as follows: (a) Those who were underweight had a BMI <18.5 kg/m2; (b) Normal, with a BMI of 18.5-22.9 kg/m2; (c) People who were overweight had a BMI of 23-24.9 kg/m2; (d) Obese people with a BMI of ≥25 kg/m2.

## Results

A total of 225 COVID-19 patients confirmed by positive RT-PCR test were included in the study. Their mean (SD) age was 45.5 (16.4) years. Almost 43% were less than 40 years old while, 18% were more than 60 years old. Males comprised more than two-thirds of the patients (68%). The BMI of half of them was within the normal range, 33.6% were overweight, and 10% were obese. The most common comorbidities were diabetes and HTN, followed by hypothyroidism, coronary artery disease, and chronic renal disease. The average blood Vitamin D level in the COVID-19 patients was 21.2 (11.1) ng/ml. Less than half of the patients (47.5%) had an appropriate Vitamin D level, 39% had Vitamin D insufficiency, and 13.5% had Vitamin D deficiency. Of the patients, 14.7% suffered from severe COVID-19, while the rest had mild to moderate disease. The mortality rate was 11% among these patients. Clinical characteristics and vitamin D status of the COVID-19 patients is given in Table 1.

**Table 1:** Clinical characteristics and vitamin D status of the COVID-19 patients (n=225) n: number of participants; BMI: body mass index; COVID-19: coronavirus disease 2019

Characteristics	n	%
Age (year)	Mean = 45.5	SD = 16.4
Age category (years)		
<40	97	43.1
41-60	87	38.7
>60	41	18.2
Sex		
Male	153	68
Female	72	32
BMI (kg/m^2^)	Mean = 24.6	SD = 4.3
BMI		
Obese	23	10.2
Overweight	76	33.8
Normal	113	50.2
Underweight	13	5.8
Alcohol intake		
No	171	75.7
Yes	54	24.3
Smoking		
No	173	76.8
Yes	52	23.2
Comorbidities		
Diabetes mellitus	25	11.1
Hypertension	25	11.1
Hypothyroidism	8	3.6
Coronary artery disease	7	3.1
Chronic kidney disease	6	2.7
Asthma	3	1.3
Hepatitis	2	0.9
Carcinoma	2	0.9
Chronic obstructive pulmonary disease	1	0.4
Vitamin D level (ng/ml)	Mean = 21.2	SD = 11.1
Vitamin D status		
Deficient	30	13.6
Insufficient	88	38.9
Optimal	107	47.5
Severity of COVID-19		
Mild	166	73.8
Moderate	26	11.5
Severe	33	14.7
Mortality of COVID-19		
No	200	88.9
Yes	25	11.1

Vitamin D deficiency was more prevalent among elderly (aged more than 60 years) patients, while insufficiency was more prevalent among younger patients (under 40 years). Besides, male patients were more likely to suffer from Vitamin D deficiency and insufficiency (17% and 40%, respectively) than their female counterparts (6% and 36%, respectively). Moreover, the prevalence of deficiency and insufficiency was more among smokers. However, these did not differ significantly according to the BMI or comorbidities of the patients. The characteristics according to Vitamin D status of the COVID-19 patients are given in Table 2,

**Table 2:** Patients profile according to Vitamin D status of the COVID-19 patients (n=225) BMI: body mass index; COVID-19: coronavirus disease 2019; n: number of participants

Characteristics	Vitamin D status			p-value
	Deficient	Inefficient	Optimal	
Age category (years)				
<40	14 (14.4)	48 (49.5)	35 (36.1)	0.016
41-60	9 (10.3)	25 (28.7)	53 (60.9)	
>60	7 (17.1)	15 (36.6)	19 (46.3)	
Sex				
Male	26 (17.00)	62 (40.5)	65 (42.5)	0.022
Female	4 (5.6)	26 (36.1)	42 (58.3)	
BMI				
Obese	5 (21.7)	8 (34.8)	10 (43.5)	0.174
Overweight	12 (15.8)	22 (28.9)	42 (55.3)	
Normal	13 (11.5)	52 (46.0)	48 (42.5)	
Underweight	0 (0.0)	6 (46.2)	7 (53.8)	
Alcohol intake				
No	19 (11.1)	66 (38.6)	86 (50.3)	0.149
Yes	11 (20.4)	22 (40.7)	21 (38.9)	
Smoking				
No	18 (10.4)	67 (38.7)	88 (50.9)	0.037
Yes	12 (23.1)	21 (40.4)	19 (36.5)	
Comorbidities				
Diabetes mellitus	6 (24.0)	8 (32.0)	11 (44.0)	0.242
Hypertension	6 (24.0)	8 (32.0)	11 (44.0)	0.242
Chronic kidney disease	2 (33.3)	1 (16.7)	3 (50.0)	0.266
Hypothyroidism	3 (37.5)	3 (37.5)	2 (25.0)	0.104
Hepatitis	1 (50.0)	0 (0.0)	1 (50.0)	0.243
Carcinoma	0 (0.0)	1 (50.0)	1 (50.0)	0.847
Coronary artery disease	2 (28.6)	3 (42.9)	2 (28.6)	0.400
Asthma	0 (0.0)	1 (33.3)	2 (66.7)	0.717
Chronic obstructive pulmonary disease	0 (0.0)	0 (0.0)	1 (100.0)	0.575

Older patients (above 60 years old) and male patients were more likely to have a severe condition than younger and female patients. Furthermore, smoking and having many comorbidities were both associated with severe illness. Serum Vitamin D levels were considerably lower in COVID-19 individuals with severe disease (mean 18.1 ng/ml versus 21.7 ng/ml in non-severe patients). Similarly, individuals who were Vitamin D deficient had a higher rate of severe illness (36.7%) than those who were not. COVID-19 mortality was also associated with older age (36.6% among the patients aged >60 years, 9% among patients aged between 40 to 60 years, and 2% among patients age <40 years), male sex (14% among males and 4% among females), smoking (19% among smokers and 9% among non-smokers), and multiple comorbidities (65% among patients with multiple comorbidities, 20% among patients with single comorbidity, and 8% among non-comorbid patients). Although blood Vitamin D levels were lower in the patients who died and mortality was greater in Vitamin D deficient individuals, the differences were not statistically significant. Table 3 gives the comparison of disease severity and mortality of the COVID-19 patients in the study.

**Table 3:** Comparison of disease severity and mortality of the COVID-19 patients (n=225) BMI: body mass index; COVID-19: coronavirus disease 2019; n: number of participants

Characteristics	Disease severity		p-value	Disease mortality		p-value
	Non-severe	Severe		No	Yes	
Age (year), Mean (SD)	43.0 (15.4)	59.8 (15.0)	0.206	43.3 (15.4)	62.8 (14.5)	<0.001
Age category (years)						
<40	94 (96.9)	3 (3.1)	<0.001	95 (97.9)	2 (2.1)	<0.001
41-60	73 (83.9)	14 (16.1)		79 (90.8)	8 (9.2)	
>60	25 (61.0)	16 (39.0)		26 (63.4)	15 (36.6)	
Sex						
Male	125 (81.7)	28 (18.3)	0.025	131 (85.6)	22 (14.4)	0.023
Female	67 (93.1)	5 (6.9)		69 (95.8)	3 (4.2)	
BMI (kg/m2), Mean (SD)	24.4 (4.3)	25.3 (4.2)	0.715	24.5 (4.3)	25.4 (4.8)	0.321
BMI						
Obese	18 (78.3)	5 (21.7)	0.107	19 (82.6)	4 (17.4)	0.202
Overweight	60 (78.9)	16 (21.1)		64 (84.2)	12 (15.8)	
Normal	102 (90.3)	11 (9.7)		105 (92.9)	8 (7.1)	
Underweight	12 (92.3)	1 (7.7)		12 (92.3)	1 (7.7)	
Alcohol intake						
No	147 (86.0)	24 (14.0)	0.634	152 (88.9)	19 (11.1)	1.000
Yes	45 (83.3)	9 (16.7)		48 (88.9)	6 (11.1)	
Smoking						
No	153 (88.4)	20 (11.6)	0.016	158 (91.3)	15 (8.7)	0.034
Yes	39 (75.00	13 (25.0)		42 (80.8)	10 (19.2)	
Comorbidity						
Multiple	6 (35.3)	11 (64.7)	<0.001	10 (58.8)	7 (41.2)	<0.001
Single	31 (79.5)	8 (20.5)		32 (82.1)	7 (17.9)	
No comorbidity	155 (91.7)	14 (8.3)		158 (93.5)	11 (6.5)	
Vitamin D level (ng/ml), Mean (SD)	21.7 (10.2)	18.1 (14.7)	0.030	21.6 (10.9)	17.5 (11.3)	0.084
Vitamin D status						
Deficient	19 (63.3)	11 (36.7)	<0.001	23 (76.7)	7 (23.3)	0.050
Insufficient	77 (87.5)	11 (12.5)		78 (88.6)	10 (11.4)	
Optimal	96 (89.7)	11 (10.3)		99 (92.5)	8 (7.5)	

Older age, multiple comorbidities, and Vitamin D deficiency were significant predictors of severe COVID-19 infection. Multiple logistic regression model demonstrates that patients age >60 years had 23 times more likely to have a severe illness (adjusted OR (aOR) 23.53, 95%CI 4.67-118.61), while those who aged between 40 to 60 years had 11 times higher risk (aOR 10.86, 95%CI 2.39-49.31). Similarly, patients with multiple comorbidities had 10 times the increased risk of severe COVID-19 (aOR 9.94, 95%CI 2.47-39.88). Vitamin D deficiency increased the risk of severe infection by almost five times (aOR 4.72, 95%CI 1.31-17.03).

In the case of mortality, only older age and male sex were revealed as significant predictors in multiple logistic regression models. Patients aged >60 years had a 35 times higher risk of mortality (aOR 35.67, 95%CI 6.003-212.01), while those aged between 40 to 60 years had seven times higher risk (aOR 7.18, 95%CI 1.319-39.16). COVID-19 mortality was five times greater in male patients than in female ones (aOR 5.66, 95%CI 1.138-28.16). COVID-19 mortality was not substantially increased by vitamin D deficiency. (aOR 3.06, 95%CI 0.740-12.72 deficiency and aOR 2.52, 95%CI 0.758-8.42 for insufficiency). Table 4 shows the predictors of severe disease and mortality of the COVID-19 patients.

**Table 4:** Predictors of severe disease and mortality of the COVID-19 patients (multiple logistic regression models) aOR: adjusted odds ratio; Ref.: reference category; COVID-19: coronavirus disease 2019

Characteristics	aOR (95% CI) for Severe disease	p-value	aOR (95% CI) for mortality	p-value
Age				
>60	23.53 (4.67-118.61)	<0.001	35.67 (6.003-212.01)	<0.001
41-60	10.86 (2.39-49.31)	0.002	7.18 (1.319-39.16)	0.023
<40	Ref.		Ref.	
Sex				
Male	3.68 (0.94-14.29)	0.060	5.66 (1.138-28.16)	0.034
Female	Ref.		Ref.	
BMI				
Obese	0.68 (0.04-10.95)	0.790	0.49 (0.030-8.24)	0.624
Overweight	0.53 (0.04-6.54)	0.623	0.28 (0.022-3.70)	0.339
Normal	0.32 (0.06-3.94)	0.374	0.18 (0.014-2.47)	0.204
Underweight	Ref.		Ref.	
Alcohol intake				
No	0.59 (0.18-1.92)	0.385	0.55 (0.150-2.03)	0.372
Yes	Ref.		Ref.	
Smoking				
No	2.33 (0.73-7.44)	0.150	2.57 (0.727-9.14)	0.143
Yes	Ref.		Ref.	
Comorbidity				
Multiple	9.94 (2.47-39.88)	<0.001	3.45 (0.821-14.51)	0.091
Single	1.04 (0.33-3.27)	0.939	0.99 (0.275-3.60)	0.996
No comorbidity	Ref.		Ref.	
Vitamin D status				
Deficient	4.72 (1.31-17.03)	0.018	3.06 (0.740-12.72)	0.122
Insufficient	1.72 (0.58-5.10)	0.325	2.52 (0.758-8.42)	0.131
Optimal	Ref.		Ref.	

## Discussion

In the current study, we discovered a significant link between a low level of Vitamin D and the risk of contracting SARS-CoV-2, supported by previous research that found Vitamin D had protective effects against respiratory distress. Additionally, a study suggested that increasing Vitamin D intake has been related to a diminished danger of upper or lower respiratory tract infections (RTIs). Vitamin D's possible role in viral infections like SARS-CoV-2 is clarified by its role in sustaining resistance and immunity. Vitamin D may also aid in the treatment of COVID-19 by preventing hypercytokinemia and the accompanying acute respiratory distress syndrome, which is generally the cause of high fatality rates [15].

Different studies from 2007 to 2020 revealed that Vitamin D had protecting effects against many acute respiratory issues, although these were of minimal quantity and lesser importance [16]. The unique link between COVID-19 risk factors and Vitamin D insufficiency includes obesity, old age, ethnicity of Asian origin or Black, and these linkages have led to the conclusion that supplementing one’s diet with Vitamin D can help one avoid or treat the illness efficiently. Moreover, it would not be wrong to say that there are chances that Vitamin D positively modifies host response to ARDS in the case of COVID-19 [17]. It does so in both phases when there is early infection by SARS-CoV-2 or an inflammatory phase [18].

In those who are neither obese nor old, there has also been a link between getting sufficient Vitamin D and having a lower COVID-19 death rate. The polymorphism of Vitamin D and its Vitamin D Binding Protein (DBP) has been discovered to have a great relationship. The DBP has three alleles, out of which amount of Vitamin D was found to be highest among DBP1-1, medium among DBP1-2, and lower among DBP2-2, in females who were white and premenopausal [17]. Furthermore, an elevated concentration of actin is detected in ARDS patients, which is one of the symptoms in COVID-19 patients, leading to angiopathy, micro-embolisms, and organ failure. Vitamin D binding protein has the function of scavenging actin to destroy it extracellularly and prevent its accumulation in the circulation. A lower level of Vitamin D binding protein in plasma shows a poor survival rate. Although DBP and actin complexes have no effects, they may also show a function similar to cytokine storm. The E-value for the outcomes linked with Vitamin D concentration and COVID-19 was determined in research. On the odds ratio scale, the result indicated minor impacts. It was determined that an odds ratio of at least 8.6 to 10.6 was required to eliminate the link between Vitamin D deficiency and ARDS in patients over the age of 65, and mortality in patients with a BMI less than 30.

Vitamin D metabolites have, for quite some time, been known to help inborn anti-viral effector components, along with enlistment of antibacterial peptides as well as autophagy [19]. Research information identifying with impacts of Vitamin D on COVID-19 is scant; however, one investigation has found that the active form of Vitamin D has an antiviral effect when tested, i.e., 1,25 dihydroxy Vitamin D in the epithelial cells of the nose that are tainted with COVID-19. It has also been appeared to control immune-pathological inflammation-causing reactions to other respiratory tract diseases. The renin-angiotensin framework that intervened with these impacts in animal testing has specific importance regarding extreme infection with SARS-CoV-2, in which the renin-angiotensin system was found to be overactive, resulting in a dismal or unfavorable prognosis [20]. Research showed that people having Vitamin D deficiency had 80% more chances to get infected, similar to our research results that show the disease severity increasing with deficient Vitamin D [21]. Out of a total of 13.6% of patients deficient in Vitamin D, 36.7% showed severity with a p-value of 0.001. While with optimal levels of Vitamin D, i.e., 47.5%, patients getting towards severity were only 10.3%.

A review consisting of many studies showed aspects of nutritional value and its status among the elderly, including impact on infection from SARS-CoV-2. There was a high prevalence rate among the elderly with undernutrition suffering from COVID-19. It showed more links with negative consequences, including higher death rates and more shifting towards intense care units. There were decreased levels of Vitamin D and other nutrients such as magnesium, albumin, zinc, etc. These resulted in more need for oxygen therapy and intense care unit transfers to cope with decreasing survival rates [22]. The study concluded that more care must be provided to the elderly population so that there is lesser malnutrition and thus more chances of survival from COVID-19. Our study showed that out of 18% (n=41) of patients with ages > 60, 17.1% (n=7) were deficient in Vitamin D. As per our study findings, a total of 5.8% (n=13) were underweight, amongst them 46.2% showed an insufficient amount of Vitamin D, and 53.8% had optimal average amount, and 0% had deficiency regardless of age.

A research study was conducted to determine the effects of Vitamin D taken six months before infection [23]. All data related to the inflammatory status, Vitamin D deficiency, and other clinical values were considered. Two groups were made, and patients were divided into having Vitamin D quantity lower than 20ng/ml and more than 20ng/ml, respectively [24]. The group with lesser levels of Vitamin D, i.e., <20ng/ml, showed decreased leukocyte (p=0.021) and hemoglobin (p=o.035) levels and higher amounts of C-reactive protein(CRP). Moreover, people who did not take Vitamin D supplementation before six months showed more chances of pneumonia than those who took Vitamin D. A range of aspects might affect DBP and levels of Vitamin D like obesity, chronic kidney and liver disease, carcinoma, CAD, asthma, diabetes, and HTN [25]. Vitamin D deficit and insufficiency were linked to illness severity and death in our research, and both were statistically significant. Diabetes and HTN, and other comorbidities, played an important role in severity and mortality, with statistically significant results among COVID-19 patients.

Another study discovered a link between vitamin D levels and parathyroid hormone levels (PTH). After validated PCR testing and diagnosis, 109 individuals ranging in age from 14 to 58 years were enrolled in the research. After eight weeks, abnormal vitamin D levels were discovered, as well as an increase in the amount of PTH. Those were the patients who needed intense care treatment; however, the low levels of Vitamin D were not linked to disease severity, i.e., lung functions and other outcomes. However, there was an abnormal relationship between Vitamin D and PTH concentrations during their phases of recovery [26].

Vitamin D can have various effects in our body, ranging from inactivating the viral pathogens by regulating the functions of antibacterial peptides, thus inhibiting the process of replication. It also activates the phagocytes, thus acting as a protective barrier for the respiratory tract. It also suppresses the process of inflammation by having a role in the making of cytokines and helper T-17 cells, thus providing a healthy environment for the proper functioning of the respiratory tract and the whole body [27].

Strengths

This was among the few studies from this region to find out the relation between Vitamin D and COVID-19. There were some case reports published but this study with enough samples give in-depth information.

Limitations

There is an ongoing debate whether infection/inflammation reduces the Vitamin D level or low level of VD makes the individual more susceptible to infection. Self-reporting of certain data like supplements, smoking, alcohol consumption should be interpreted with care, To confirm these findings, more clinical trials and multi-center studies are required. 

## Conclusions

The level of vitamin D in the body has been linked to the severity of illness. Vitamin D deficiency and insufficiency were found to be prevalent in 13.6% and 38.9% of the participants in the current study, respectively. Our findings add to a stream of research suggesting that a patient's Vitamin D insufficiency is a predicted risk factor for a worse COVID-19 critical illness and mortality. More study is needed to determine when and whether vitamin D supplementation in the presence of vitamin D deficient persons in society affects the outcome of a COVID-19 episode. While substantial randomized controlled trial (RCT) data is needed to support the use of Vitamin D in the care of COVID-19, clinicians should continue to treat Vitamin D shortage and insufficiency in COVID-19 patients until that time comes, as there are no adverse effects.
